# Impact of summer drought on isoprenoid emissions and carbon sink of three Scots pine provenances

**DOI:** 10.1093/treephys/tpw066

**Published:** 2016-11-28

**Authors:** M. Lüpke, M. Leuchner, R. Steinbrecher, A. Menzel

**Affiliations:** 1 Ecoclimatology, Technische Universität München, Hans-Carl-von-Carlowitz-Platz 2, 85354Freising, Germany; 2 Institute for Advanced Study, Technische Universität München, Lichtenbergstraße 2a, 85748Garching, Germany; 3 Springer Science+Business Media B.V., Van Godewijckstraat 30, 3311GX Dordrecht, The Netherlands; 4 Karlsruhe Institute of Technology KIT, Institute of Meteorology and Climate Research Atmospheric Environmental Research (IMK-IFU) , Kreuzeckbahnstraße 19, 82467Garmisch-Partenkirchen, Germany

**Keywords:** BVOC, chemotypes, drought stress, provenances, Scots pine

## Abstract

Scots pine (*Pinus sylvestris* L.) provenances cover broad ecological amplitudes. In a greenhouse study, we investigated the impact of drought stress and rewetting on gas exchange for three provenances (Italy: Emilia Romagna; Spain: Alto Ebro; Germany: East-German lowlands) of 2-year old Scots pine seedlings. CO_2_, water vapour and isoprenoid exchange of stressed and control trees were quantified with a four-chamber dynamic-enclosure system in the controlled environment of a climate chamber. The three provenances showed distinct isoprenoid emission patterns and were classified into a non-Δ^3^-carene, with either high α-/β-pinene or β-myrcene fraction, and a Δ^3^-carene dominated type. Isoprenoid emission rates, net-photosynthesis and transpiration were reduced during summer drought stress and significantly recovered after rewetting. A seasonal increase of isoprenoid emission rates towards autumn was observed for all control groups. Compared with the German provenance, the Spanish and Italian provenances revealed higher isoprenoid emission rates and more plastic responses to drought stress and seasonal development, which points to a local adaptation to climate. As a result of drought, net carbon uptake and transpiration of trees was reduced, but recovered after rewetting. We conclude from our study that Scots pine isoprenoid emission is more variable than expected and sensitive to drought periods, likely impacting regional air chemistry. Thus, a provenance-specific emission assessment accounting for reduced emission during prolonged (summer) drought is recommend for setting up biogenic volatile organic compound emission inventories used in air quality models.

## Introduction

Biogenic volatile organic compounds (BVOCs) are emitted into the atmosphere by many biomes. BVOCs contribute to various atmospheric processes such as tropospheric ozone chemistry or formation of secondary organic aerosols. In the northern hemisphere many terrestrial biomes are dominated by conifer species that are known for synthesizing and emitting isoprenoids, with monoterpenes (MTs) as a prominent group within the BVOCs (Kesselmeier and Staudt 1999). Identified ecological functions of MTs are, for example, communication processes between plants, defence against herbivores or fungi, and attraction of predators or pollinators (Langenheim 1994) besides their impact on chemical processes in the atmosphere (Atkinson and Arey 2003).

Monoterpenes are emitted into the atmosphere by different processes, such as diffusion through the cell wall and stomata (Niinemets et al. 2004) or damage of wood and leaf structures with resin- containing structures with MT pools, e.g., by insects (Klepzig et al. 1995, Achotegui-Castells et al. 2013), wind (Haase et al. 2011) and other mechanical influences (Schade et al. 2011). The emitted amount of MTs is limited by the current storage in pools and the actual synthesis capacity (Niinemets et al. 2004, Ghirardo et al. 2010). Both source types are driven by light and temperature (Shao et al. 2001, Dindorf et al. 2006). Synthesis and storage of MTs and hence actual emission rates can be further influenced by leaf and tree age (e.g., Komenda 2002, Thoss et al. 2007), water and nutrient availability (Lerdau et al. 1995, Blanch et al. 2009), and seasonality (Bäck et al. 2005, Hakola et al. 2006). Therefore, abiotic and biotic stresses (Loreto and Schnitzler 2010, Spinelli et al. 2011) can either decrease emissions of MTs, e.g., through reduced photosynthesis by drought (Šimpraga et al. 2011), or an enhanced production of MTs can be induced as a result of leaf/wood destruction by insects in order to attract predators of feeding insects or as defence against herbivory through rupture of resin structures. Since the scales of stress differ in their duration and severity (Niinemets 2010), short-term stress can induce other emission schemes. These differ compared with long and/or severe stress by compound composition and amount. Also, the recovery to normal emission levels after different stress events can vary.

The emitted MTs are mixed into the atmosphere where they are either deposited as dry or wet matter (Schade et al. 2011, Niinemets et al. 2014), photolysed or can react with other oxidants to form secondary compounds (Atkinson and Arey 2003). Under normal atmospheric conditions MTs have a short lifetime ranging from several minutes to several hours (Guenther et al. 1995, Atkinson and Arey 2003, Perakyla et al. 2014) due to oxidation processes with OH and NO_3_ radicals, and O_3_ (Atkinson and Arey 2003) forming new compounds. Yet, these lifetimes largely differ between various MT compounds and their oxidation partner (Atkinson and Arey 2003). These processes lead to build-up or destruction of ozone (Atkinson and Arey 2003) and the formation of biogenic secondary organic aerosol (SOA) (e.g., Plewka et al. 2006, Perakyla et al. 2014). Biogenic SOAs result in a negative radiative forcing and cause a cooling effect in the atmosphere (Scott et al. 2014); furthermore, they act as cloud condensation nuclei (Pierce et al. 2012). On the other hand the production of ozone can lead to a positive radiative forcing (Stevenson et al. 2013) with further stress for the biosphere (Felzer et al. 2007). Most of the BVOC-mediated impacts on the atmosphere can enhance atmospheric warming, causing a positive (reinforcing) feedback on the biosphere and its emissions (Kulmala et al. 2004).

The amount of these BVOC emissions is dependent on abiotic and biotic factors, resulting in a wide range of reported emission rates. Current models (e.g., Arneth et al. 2008, Guenther et al. 2012) estimate around 33–160 Tg year^−1^ of MTs and 400–600 Tg year^−1^ of isoprene that are currently emitted globally by the biosphere into the atmosphere. The wide range of reported estimates may partly be attributed to the uncomplete integration of processes affecting the isoprenoid emissions of plants (Peñuelas and Staudt 2010), such as stress by drought (Grote et al. 2010) changing emission patterns with season (Staudt et al. 2003) and varying emissions within species resulting from different chemotypes (Smolander et al. 2014).

Scots pine (*Pinus sylvestris* L.) is a widely distributed conifer tree species growing from south-western Europe to Scandinavia and Siberia and from near sea level up to more than 2000 m above sea level in the south. This species is characterized by various local provenances showing different morphology as adaptation to local conditions (Oleksyn et al. 1998). Already in the 1970s different compositions of MTs in needles were used to identify chemotypes (Tobolski and Hanover 1971) and to distinguish provenances. This difference in isoprenoid composition was also observed within local populations (e.g., Bäck et al. 2012, Yassaa et al. 2012, Kannaste et al. 2013) and between different plant compartments such as leaves and bark (e.g., Sallas et al. 2003, Ghirardo et al. 2010). Scots pine contains various isoprenoids such as isoprene, different MTs and also sesquiterpenes (e.g., Sallas et al. 2003, Tarvainen et al. 2005, Holzke et al. 2006*b*, Kannaste et al. 2013). Their composition and amount change over the season with ongoing plant development (e.g., Bäck et al. 2005, Tarvainen et al. 2005, Räisänen et al. 2009). Isoprenoid emissions were shown to be as quite complex, since they can derive from pools in bark/stem and leaves mainly driven by temperature as well as from de novo/photosynthetically related synthesis driven by fast carbon turn (e.g., in leaves, young bark) (Shao et al. 2001, Ghirardo et al. 2010). For de novo emissions it is known that (i) the ratio between pools and de novo emission differs between compounds (Steinbrecher et al. 1999, Shao et al. 2001, Ghirardo et al. 2010); (ii) synthesis of MT still takes place under dark conditions, probably due to stored isoprenoid precursors (Shao et al. 2001); and (iii) de novo synthesized MTs of Scots pine are stored in pools (Ghirardo et al. 2010). Ongoing climate change and increasing extreme events have already forced several provenances of Scots pine to the margins of their climatic envelope (Reich and Oleksyn 2008). Thus, although the wide distribution of Scots pine has led to specific adaptations (Oleksyn et al. 1998), e.g., root to leaf ratio or needle length, increased extreme events such as drought might have triggered local diebacks (e.g., Rebetez and Dobbertin 2004, Galiano et al. 2010). The response to drought stress of those marginal provenances has been indicated by a wide range of changes in biomass production and phenological development (Taeger et al. 2013*a*, 2013*b*). In contrast, isoprenoid emissions of different Scots pine provenances under drought stress events have not been investigated so far. In order to understand different adaptation strategies to projected climate change we investigated three carefully selected Scots pine provenances in order to test the following key hypotheses:

- Chemotype patterns of Scots pine seedlings differ among and within provenances, but individual fingerprints remain also under different treatments.
- Scots pine isoprenoid emissions rates change during and after long-term drought stress, but can recover to non-stressed (normal) levels after rewetting.
- Provenances of Scots pine show different responses to drought stress and during recovery.

To analyse the effects of provenance and drought on Scots pine, we used a self-built dynamic-enclosure system and investigated tree gas exchange applying a summer drought followed by a 6-week recovery phase, in which the water supply was optimal for both the control and the stressed group.

## Materials and methods

### Experimental design

From a previous study by Taeger et al. (2013*a*) seedlings of 10 Scots pine provenances were available for our experiment. Three provenances were selected based on the following criteria: (i) low genetic relationship, (ii) different levels of drought adaptation and (iii) growing at margins of the distribution area. Therefore, we used 2-year-old trees of Scots pine provenances grown from seeds from Spain (Alto Ebro, 42°59′ N03°17′W) (ESP), Italy (Emilia Romagna, 44°30′N 10°27′E) (ITA) and Germany (East-German lowlands/Brandenburg, 53°04′N 13°29′E) (GER) in this study.

One-year-old seedlings grown in a seedling nursery were planted in autumn 2012 into 2-l pots with organic substrate (Klasmann-Deilmann GmbH, Geeste, Lower Saxony, Germany) of type ‘Basismischung Bayer. Staatsforsten AöR’ containing turf and pine bark. According to the manufacturer's specification this substrate had a dry mass of <10%, a maximum water capacity of 75–80% and 10–15% air capacity. Nutrient contents were 210 mg N l^−1^, 240 mg P_2_O_5_ l^−1^, 270 K l^−1^ and 120 mg Mg l^−1^ at a pH value of 5.2. Additionally, a soil moisture retention curve was established to identify the permanent wilting point (PWP) of pF 4.2 at a volumetric soil water content (SWC) of 0.12 m^3^ m^−3^ (± 0.02 m^3^ m^−3^) (see also Supplementary Figure S1 available at *Tree Physiology* Online for pF-curve and Pearson et al. 2013) and field capacity ranging up to 0.40 m^3^ m^−3^.

The potted trees were grown in a greenhouse and watered by a drip irrigation system (NETAFILM, Tel Aviv, Israel). Tests showed a variation between single drippers of ∼5% which corresponded to the manufacturer's data. We randomly selected 12 trees per provenance out of the large-scale drought greenhouse experiment, however only individuals smaller than 50 cm were used in order to fit to our custom-built plant enclosure system. Prior to our investigation all trees were exposed to a mild spring-drought phase from 22 March 2013 to 10 June 2013 followed by a first rewetting phase. Compared with non-stressed trees growth was reduced but during the first rewetting stressed trees showed needle growth and diameter increase. These trees were therefore better suited for the experiment due to their size and prior drought acclimation. Half of these trees additionally experienced a 6-week summer drought phase lasting from 11 July 2013 to 22 August 2013. Drought stress was regulated through the dripping system targeting at SWC oscillating around 0.12 m^3^ m^−3^. The other half was used as a control group under normal irrigation, i.e. watered in intervals between one and three days with 100–150 ml (total amount of 3500 ml) depending on meteorological conditions in the greenhouse. In contrast, drought- stressed seedlings were watered six times with a total amount 725 ml and the last watering of 250 ml at 15 August 2013 (see also Supplementary Figure S1 available at *Tree Physiology* Online).

### Enclosure system

The gas exchange including MT emissions of the trees was investigated with a separate enclosure system placed within a climate chamber to guarantee controlled measurement conditions (see Supplementary Figure S3 available at *Tree Physiology* Online for a photo of the enclosure system). The four-chamber custom-built dynamic-enclosure system was designed following suggestions by Ortega and Helmig (2008) and Niinemets et al. (2011). In order to achieve high inertness and low reaction surfaces we used fluoroelastomer (FKM) rubber or polytetrafluoroethylene (PTFE) bands as sealing material, perfluoroalkoxy alkanes (PFA) plastic as tubing and stainless steel for fittings and valves. The 30-l chambers (diameter 26.5 cm and height 60 cm) consisted of a polyvinylidene difluoride plastic bag (SUPELCO, Supelco Inc, Bellefonte, Pennsylvania, USA), fixed on a stainless steel flange (BEVAB GmbH, Bergisch Gladbach,North Rhine-Westphalia, Germany) mounted on two high-grade aluminium plates on a frame. These plates fit snugly around the stem, separating the measured tree crown from the rest of the plant. To insure a good seal, the stem was wrapped with PTFE tape. The air volume of each chamber was exchanged with a flow of 15 standard litres (1 l at 0 °C, 1013 hPa) per minute (l_n_ min^−1^), controlled by mass flow controllers (SMART4S GSC, Vögtlin Instruments AG, Aesch, Basel-Landschaft, Switzerland). Background VOCs and other contaminants were scrubbed from the inlet air by first purifying the pressurized air with a zero-air generator (AERO40LS, Peus-instruments GmbH, Gaggenau, Germany) followed by a 10-l tank of activated charcoal filter (VWR International GmbH, Darmstadt, Hessia, Germany). The dry air was then humidified in a bubbling vessel with ultra-purified water. CO_2_ levels in air fed to the chambers were set to 400 µmol mol^−1^ by adsorbing first all CO_2_ with soda lime and then re-adding purified CO_2_ (purity ≥99.995%, Westfalen AG, Germany) with a mass controlled flow (SMART6 GSC, Vögtlin Instruments AG, Aesch,Basel-Landschaft, Switzerland). By means of an infrared gas analyser (CIRAS-2 DC, PP Systems International, Inc., Amesbury, Massachusetts, USA), absolute inlet air CO_2_ and humidity levels were monitored continuously. Differences in CO_2_ and H_2_O molar fractions between plant-enclosure inlet and outlet were determined over 5 min for each chamber by continuously switching between the four enclosures. Net photosynthesis *P*_N_ and transpiration rates *T*_R_ (see Supplementary Eq. S1 and S2 available at *Tree Physiology* Online) of the respective trees were then calculated from the molar fraction differences between chamber inlet and outlet according to von Caemmerer and Farquhar (1981) based on leaf area. Isoprenoids were sampled automatically from the outlet air of each of the four plant chambers for assessing isoprenoid emissions. Adsorption tubes were used for pre-concentrating BVOC in the enclosure (see Isoprenoid sampling and analysis).

Environmental settings, sampling flow rates and collection times as well as chamber flow parameters were continuously monitored and controlled. In each chamber, surface temperatures of two needles were acquired with thermocouples (5TC-TT-KI-40-5M, Omega Engineering Limited, Manchester, England, UK) and air temperature and relative humidity in the closure were measured by a combined sensor (BB SENSORS, B+B Thermo-Technik GmbH, Donaueschingen, Baden-Wuerttemberg, Germany Germany). A PAR sensor (SKL 2610, Skye Instruments Ltd, Llandrindod Wells, Wales, UK) measured photosynthetic active radiation. For illuminating the climate chamber, a mixture of white lamps, Lumilux® Cool White and plant lights, Fluora® (OSRAM GmbH, Munich, Bavaria, Germany), was used (ratio 2:1). An additional LED lamp (BloomPower white360, spLED GmbH, Flensburg, Schleswig-Holstein, Germany) was installed above the chamber to enhance climate-chamber illumination from 240 to 600 µmol PAR m^−2^ s^−1^ generating a light climate closer to summer conditions.

The parameters for the climate chamber harbouring the plant enclosure and sampling system were set to a 12-h day with a constant air temperature of 24 °C and a relative air humidity of 60%. Light levels were increased in hourly steps from 0 to 50, 50 to 100, 100 to 250, and finally from 250 to 600 µmol PAR m^−2^ s^−1^ to simulate a night–day transition. The last light increase was achieved by the LED illumination.

### Sample design

Isoprenoids were sampled on nine days at the end of the drought treatment from 13 August 2013 to 22 August 2013 (no measurement at 15 August  due to watering) and on nine days after the recovery phase of 6 weeks with regular watering from 8 October 2013 to 16 October 2013. For both sampling periods, the same individuals were monitored to check for recovery and seasonal effects. In total, 36 trees were used. For each of the three provenances two trees per treatment (stressed, control) were investigated simultaneously and this setting was replicated three times, resulting in six trees sampled per provenance and treatment as shown in Table 1.

**Table 1.** Sample design (provenances: GER = German, ESP = Spanish, ITA = Italian).

Sample periods in 2013	Treatment per day	Provenance on day of the sample period									
		1	2	3	4	5	6	7	8	9	10
Drought (13– 22 August)	Two drought-stressed + two well-watered individuals	GER	ESP	–	ITA	GER	ESP	ITA	GER	ESP	ITA
Rewetting (8–16 October)	Identical individuals used as for drought, however, drought stressed ones re-watered	GER	ESP	ITA	GER	ESP	ITA	GER	ESP	ITA	–

On each of the sampling days in the afternoon, four individuals of the same provenance (two from control and two from the drought treatment) were installed into the four chambers of the sampling system. On the following sampling day, we switched to the next provenance (see Table 1). By this rotation, a set of trees of each provenance was measured at the beginning, the middle and the end of each sampling period, respectively, to account for small seasonal effects within each sample period. Each tree had an acclimation time of 10–12 h before start of sampling to acclimate to chamber conditions and to reduce possible effects of mechanical stress on the plants during installation. Emissions were sampled twice during night-time, beginning at 03:00 and 04:30 h CEST, and twice during daytime, beginning at 11:00 and 12:30 h, each for 75 min.

Before and after mounting potted trees into the enclosure system, SWC was measured with a mobile time-domain reflectometry (TDR) probe (UGT GmbH, Müncheberg, Brandenbug, Germany, Germany) and additionally each pot was weighed. Control trees in summer were also checked for sufficient SWC levels (above wilting point) before installation. At the end of the experiment, all biomass from plant parts within the chamber was harvested. Mass of stems and needles was determined after drying the material for 48 h at 60 °C. The area-to-mass ratio was determined by weighing and scanning 10 randomly selected needle pairs for each individual tree. Needle area was calculated with I imageJ software (Schneider et al. 2012). The area-to-mass ratio was used to convert total needle dry mass to total tree leaf area. Since no needle growth between both summer and autumnal sample periods was observed, the same leaf area was used for both periods in the calculations.

### Isoprenoid sampling and analysis

Isoprene and MTs were sampled onto inert, carefully conditioned (see below) stainless steel adsorption tubes (CAMSCO Inc., Houston, Texas, USA) filled with a two-bed substrate of 40 mg Carbotrap® 5TD (60/80 mesh) and 70 mg Tenax® TA (60/80 mesh). The adsorption tube sampling gas flow was set at 150 ml_n_ min^−1^ by a downstream-installed mass flow controller (SMART6 GSC, Vögtlin Instruments AG, Aesch, Basel-Landschaft, Switzerland) in front of a vacuum pump (607CD22, Gardner Denver Thomas GmbH, Fürstenfeldbruck, Bavaria, Germany). Using this sample gas flow and a sampling time of 75 min, a breakthrough of target compounds was not observed as indicated by a complete recovery of all compounds of a 16-component gas standard in the first of two adsorption tubes arranged in series (single compound amount fractions of ∼2 nmol mol^−1^; NPL, Teddington, England, UK). Prior to each sampling an internal standard of Δ²-carene (SIAD Austria GmbH, St. Pantaleon, Upper Austria, Austria) with 87 nmol mol^−1^ was added with 25 ml_n_ min^−1^ for 2 min onto each clean tube. After each measurement the tubes were directly analysed by gas chromtograph (GC) with flame ionization detectior (FID) and mass spectrometer (MS).

For analysis with the GC-FID/MS, each sample tube was dry purged for 3 min and then desorbed with helium (purity >99.999% , Westfalen AG) and scrubbed by a gas purging trap (SGE, Ringwood, Victoria, Australia) for 10 min at 280 °C and 50 ml_n_ min^−1^ with an ATD650 thermal desorber (Perkin Elmer Inc., Waltham, Massachusetts, USA). The desorbed analytes were pre-focused in a cold trap (Air Monitoring©, Perkin Elmer Inc., Waltham, Massachusetts, USA) at −25 °C with no inlet split and then heated rapidly at 40 °C s^−1^ to 300 °C. With a hold time of 10 min at 300 °C the analytes were transferred over a 255 °C heated-glass transfer line into the GC unit with a column flow of 1.5 ml min^−1^ and an outlet split of 2 ml min^−1^. After the GC analysis, the tubes were automatically conditioned at 310 °C for ∼20 min with a helium flow of 50 ml min^−1^. Clean tubes were sealed with air-tight end caps (CAMSCO Inc., Houston, Texas, USA) and stored at room temperature until further use.

The analytes were separated in a Clarus® SQ8 GC-MS/FID system (Perkin Elmer Inc., Waltham, Massachusetts, USA) with an Elite-5ms column (250 µm i.d., 30 m, 95% methylpolysiloxane, 5% phenyl, Perkin Elmer Inc., Waltham, Massachusetts, USA) at 1.5 ml min^−1^ with the following temperature programme: 4 min hold at 40 °C, 15 °C min^−1^ up to 100 °C, then 5 °C min^−1^ up to 240 °C and a final hold of 4 min. The mass spectrometer was set to electronic ionization mode at 70 eV with SIFI mode (single ion and full scan) at an inlet temperature of 220 °C and a source temperature of 230 °C. Single ion mode was set on the main mass fragments (*m*/*z*) 43, 68, 93 and 119 to quantify target compounds isoprene and the MTs α- and β-pinene, β-myrcene, Δ³-carene, *p*-cymene, *cis*-β-ocimene and 1,8-cineole. *Cis*-β-ocimene and 1,8-cineole were co-eluted at the same retention time and were handled as one compound. A 16-component gas standard (NPL) with single compound amount fractions of ∼2 nmol mol^−1^ containing the target compounds was used for calibration. In addition, Δ²-carene was used as an internal standard to compensate for the impact of system fluctuations on calculated target compound amount fractions. Full scan mode was used to verify detected compounds and identify additional compounds in the sample air by the NIST 08 database (Stein 2008). The detection limits of the compounds for one desorbed sample tube were in the range of 0.007 to 0.024 nmol mol^−1^, depending on the compound. Values below detection limit were either handled as zero in statistical group comparison tests and clustering or as not applicable for linear models, calculations of means.

Emission rates (in nmol m^−2^ projected leaf area s^−1^) were calculated following Eq. (1) (according to Niinemets et al. 2011):

(1) $$E=\left(\chi_{\mathrm{out}}-\chi_{\mathrm{in}}\right)F_{m}{A_{\mathrm{Leaf}}}^{-1},$$

where *χ_in_* is the amount of each quantified compound (in nmol mol^−1^) in the inlet air of each chamber and *χ_out_* the compound concentration in air downstream of the chamber derived from the sampled tube. *F_m_* is the molar air flow per second through the chamber determined by the mass flow controllers. *A*_*Lea*f_ (in m²) is the leaf area of the tree measured in the chamber.

Furthermore, water vapour build-up through plant transpiration within the chambers had to be considered in gas-exchange calculations (see Niinemets et al. 2011). Thus, the emission rate was corrected by the transpiration rate *T*_R_ (in mol m^−2^ s^−1^) (see Supplementary Eq. S1 available at *Tree Physiology* Online) and under the assumption of an inlet isoprenoid concentration of zero, which was tested by a blank chamber test. This results in Eq. (2):

(2) $$E=\chi_{\mathrm{out}}F_{m}{A_{\mathrm{Leaf}}}^{-1}+\chi_{\mathrm{out}}F_{m}T_{R}.$$

For standardizing the isoprenoid emissions, the Guenther 93 algorithm (Guenther et al. 1993) was applied:

(3) $$E_{s}=E(e^{\beta \left(T_{\mathrm{leaf}}-298.15K\right)}){}^{-1},$$

with a *β*-value of 0.09 and a standard temperature of 298.15 K (25 °C). By this correction, thermal influences on the emission rates were compensated. Correction of light was neglected since all trees had identical light conditions during daytime sampling.

### Statistical analysis

In total, 288 BVOC samples were taken during both sample periods. Six of the 288 samples had to be discarded due to contamination and system faults. Out of the valid samples mean daytime and night-time emission rates were derived for each tree. In most cases two samples for daytime and night-time each were available. The data were processed and analysed in R Version 3.1.1 (R Development Core Team 2014) and plots were made with ‘ggplot2’ (Wickham 2009). The Mann–Whitney test was used to test for group differences between treatment, provenance, day–night and sample periods. Statistical significance was accepted with a *P*-value <0.1.

Linear models checked the dependency of *E*_*S*_, *T*_R_ and *P*_N_ on soil water content with possible interaction of sample periods (seasonal signal), time of day and provenance. Model selection was done by a stepwise (both directions) reduction of input parameters by the Akaike information criterion (AIC) and fine selection of input explanatory variables and interaction with SWC within a delta AIC of 2 by hand. The dependent variables were logarithmized to improve model quality. Effect plots and interaction were extracted by the R package ‘effects’. The effect plots show fitted values of any explanatory variable (including related lower-order terms) from the selected model, holding all other model variables constant that have no interaction with this variable. These fitted values show the ‘effect’ size of this variable on the modelled responses (in this study *P*_N_, *T*_R_ and *E_S_*).

In order to classify the chemical composition of each tree's emission into chemotypes, the PAM clustering method was used. Clusters were selected by partitioning the data into k clusters around medoids. The input data consisted of the mean relative contribution of compounds within each tree individual at day-/night-time and drought/rewetting. The optimal number of clusters was first determined by their silhouette size, which ranged between three and eight clusters per sample period and day-/night-time. However, in order to keep enough samples within each cluster and for a better comparison between both sample periods and times, four clusters were always used.

## Results

Both sample periods were completed within a time frame of 9–10 days. Each tree typically stayed in the chamber system for ∼20 h for a measurement cycle in which ecophysiological parameters including isoprenoid emission for stressed and control trees were assessed.

### Photosynthesis and transpiration

**Figure 1.**
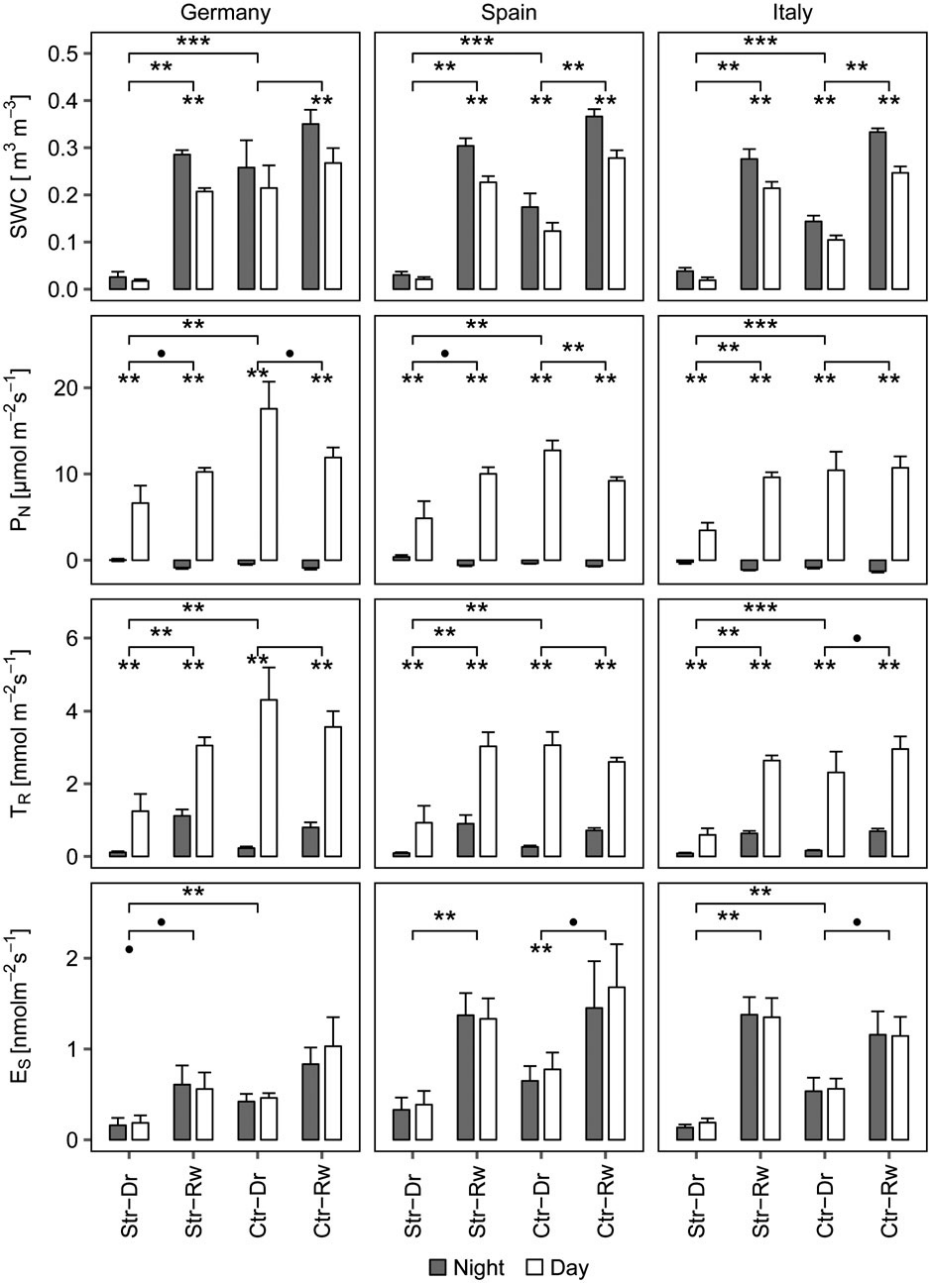
Overview of mean environmental, ecophysiological and BVOC parameters for the three provenances separated by treatment and experiment (Str = stressed, Ctr = control, Dr = drought, Rw = rewetting), and respective time (light = day, grey = night) in conjunction with selected group statistics. Bars represent the mean daytime or night-time values and error bars the standard error of each treatment group and experiment. Asterisks over bars and brackets represent significant results (***P < 0.01, **P < 0.05, •*P* < 0.1) of the group comparisons in three levels, the lower level is showing differences between day and night (Mann–Whitney, paired), mid-level differences between sample periods for control and stressed groups (Mann–Whitney, paired) and top-level differences between stressed and control group during the drought (Mann–Whitney, non-paired). *N* = 6 for each group, experiment and time. Further group statistics can be found in Supplementary Tables S1 and S2 available at *Tree Physiology* Online. Volumetric soil water content (SWC), transpiration rate (*T_R_*), net photosynthesis rate (*P_N_*) and total emission rate (*E_S_*) are normalised to 25 °C by the Guenther 93 algorithm.

Results of the experiment on drought and rewetting are shown in Figure 1 comprising mean transpiration rates *T*_R_, net photosynthesis *P*_N_ and volumetric soil-water content SWC, as well as total isoprenoid emission rates *E*_*STotal*_ (see Total isoprenoid emission) of each provenance with their respective standard error. Significant results of the group comparison between day and night, stressed and control, drought and rewetting are shown with asterisks (see Supplementary Tables S1 and S2 available at *Tree Physiology* Online for additional group comparisons).

#### Drought application

The first sampling was conducted during the last two weeks of the 6-week summer drought application. For all provenances SWC was significantly lower for stressed groups compared with control groups (see Figure 1). Regular watering ensured a clear SWC difference between both groups (see Supplementary Figure S1 available at *Tree Physiology* Online for the general water regime in the experiment), where the stressed trees had 725 ml and the control ones 3500 ml water in total. However, due to hot summer conditions in the greenhouse, several trees of the control group also had reduced SWC nearly reaching the PWP (0.10–0.14 m^3^ m^−3^). Soil water content of stressed trees ranged between 0.02 and 0.04 m^3^ m^−3^ (Figure 1), and the SWC of the control trees ranged from 0.11 (ITA) to 0.21 m^3^ m^−3^ (GER). However, due to the interval watering on 15 August, some trees of the stressed group received water above the PWP, but still showed low SWC. German control trees had a significant higher daytime SWC than Italian control trees. Soil water content differences between night and day (pre- and post-sampling) were significant only for the Spanish and Italian control groups. If not specifically mentioned, daytime mean values will from this point on.

Transpiration rates (*T*_R_) differed significantly between the stressed and the control groups of all provenances (see Figure 1). Lowest *T*_R_ among the stressed groups was observed for the Italian provenance (0.59 mmol m^−2^ s^−1^) and the highest for the German provenance (1.25 mmol m^−2^ s^−1^), whereas the respective control groups showed three to four times higher *T*_R_ ranging from 2.40 (ITA) to 4.31 mmol m^−2^ s^−1^ (GER) (Figure 1). The TR in the German control group was significantly lower than the Italian group.

Leaf temperature (*T*_Leaf_) was significantly lower in the control groups by an average of 2 °C with only small variations between the provenances ITA (stressed 28.5 °C, control 26.4 °C), ESP (stressed 28 °C, control 25.6 °C) and GER (stressed 27.9 °C, control 26 °C) (see also Supplementary Table S2 available at *Tree Physiology* Online).

Significantly lower photosynthesis rates (*P*_N_) between treatments were observed for all stressed provenances ranging from 3.45 (ITA) to 6.62 µmol m^−2^ s^−1^ (GER). Control groups exhibited higher *P*_N_ ranging from 10.71 (ITA) to 17.56 µmol m^−2^ s^−1^ (GER) (see Figure 1).

#### Recovery after rewetting

Recovery of the plants was measured after 6 weeks of rewetting. Using control plants, investigations may elucidate a seasonal effect. No trees were lost during the drought application and thus all prior investigated individuals were used in the measurements after rewetting. Loss of current-year needles was not observed during the experiment, but previous years’ needles were already thrown off before the drought sampling period.

SWC after rewetting ranged from 0.21 (stressed, GER) to 0.28 m^3^ m^−3^ (control, ESP, see Figure 1). Typically, SWC increased significantly after rewetting for the stressed groups by a factor of 5–10 and for the control groups by a factor of 1.5–2. Significant differences between day and night were detected for all groups.

After rewetting, *T*_R_ of control and stressed groups ranged between 2.60 and 3.40 mmol m^−2^ s^−1^ and did not differ significantly. *T*_R_ of the stressed groups increased significantly from drought to rewetting by a factor of 2–4, up to 3.05 mmol m^−2^ s^−1^ for the Spanish and German and up to 2.69 mmol m^−2^ s^−1^ for the Italian provenance (Figure 1).

*T_Leaf_* of the stressed groups at day was significantly reduced from drought to rewetting, decreasing by ∼2 °C to 26 °C on average.

Daytime *P*_N_ showed no significant difference between stressed and control trees (see Figure 1). A provenance effect could only be detected between the Spanish and German control groups. For stressed groups *P*_N_ significantly increased by a factor of 2–3 from drought to rewetting, up to 10.2 µmol m^−2^s^−1^, whereas the control groups decreased significantly their daytime *P*_N_ rates for the German and Italian provenances.

#### Modelling of gas exchange

**Figure 2.**
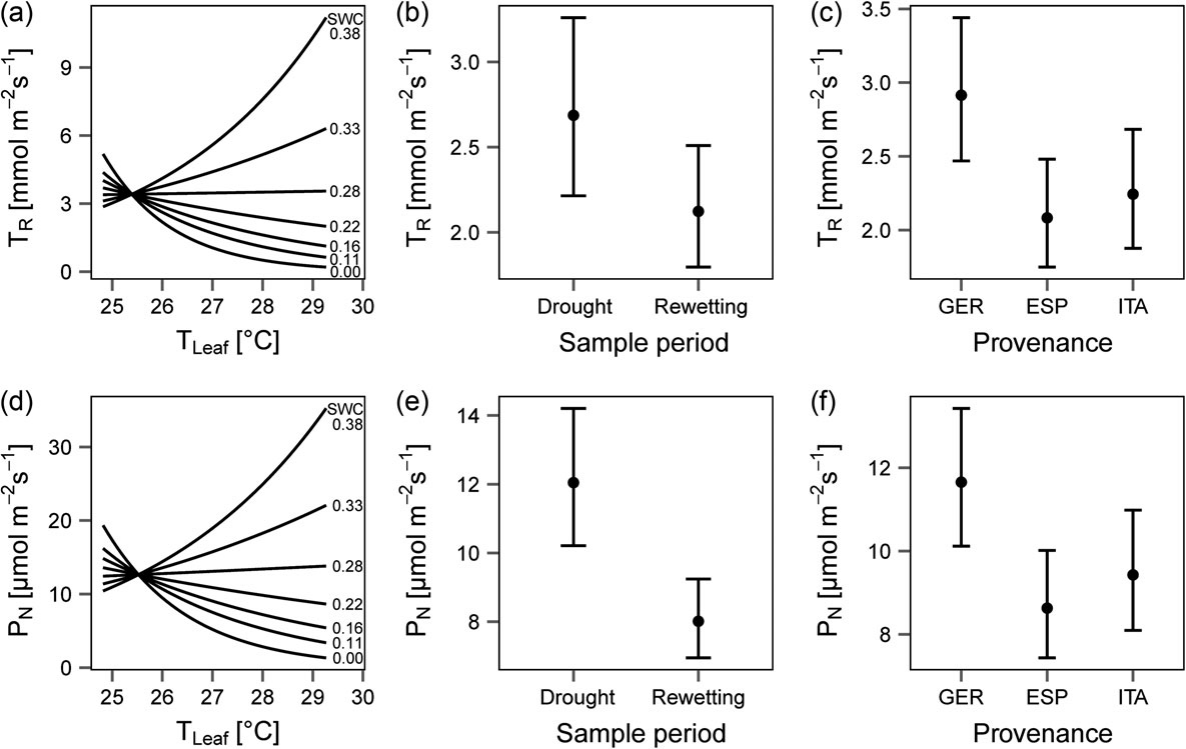
Effect plots of the linear model for daytime evapotranspiration rates (*T_R_*) (top row) and daytime net photosynthesis rates (*P_N_*) (bottom row). Panels display the significant effects of (a, d) *T*_*Leaf*_ and SWC interactions (see numbers at end of each line), (b, e) sample period and (c, f) provenance. Error bars represent the standard error. Confidence intervals in plots (a) and (d) are not presented for readability.

Impacts of SWC, drought and rewetting as well as provenance on *P*_N_ and *T*_R_ were determined by linear models with adjusted *R*² of 0.81 for *T*_R_ and 0.77 for *P*_N_ (see Supplementary Table S5 available at *Tree Physiology* Online for used model input variables). Figure 2 displays the resulting effect sizes of the significant factors for *P*_N_ and *T*_R_ (other non-interacting variables were kept constant, see Statistical analysis). Night-time measurements were excluded from this modelling approach since *P*_N_ and *T*_R_ were naturally low.

Figure 2a and d shows the effect of *T*_*Leaf*_ interacting with SWC on *P*_N_ and *T*_R_. Here, with SWC >0.28 m^3^ m^−3^ P_N_ and *T*_R_ increased with *T*_*Leaf*_, for lower SWC *P*_N_ and *T*_R_ decreased with *T*_*Leaf*_. Furthermore, the seasonal effect decreased *P*_N_ by 4 µmol m^−2^ s^−1^ (Figure 2e) and *T*_R_ by 0.56 µmol m^−2^ s^−1^ (see Figure 2b). A provenance effect was observed for the German trees with higher *T*_R_ and *P*_N_ than the other two provenances (Figure 2c and e).

### Total isoprenoid emission

Drought treatment led to significantly lower average standardized total quantified isoprenoid emission rates (*E*_*STotal*_) of 0.19 nmol m^−2^ s^−1^ for stressed trees of the German and Italian provenance (see Figure 1). This resulted in a emission reduction of ∼70% compared with the control groups with 0.46 nmol m^−2^ s^−1^ (GER) and 0.60 nmol m^−2^ s^−1^ (ITA), respectively. In contrast, *E*_*STotal*_ of the Spanish provenance ranged between 0.39 nmol m^−2^ s^−1^ for the stressed and 0.78 nmol m^−2^ s^−1^ for the control group (difference not significant).

After rewetting, *E_S Total_* did not differ significantly between the control and former drought group (Figure 1), similar to the other ecophysiological parameters. However, the formerly drought stressed plants showed a strong and significant increase in *E_S Total_* after rewetting, by a factor of 3 for the German and Spanish and by a factor of 7 for the Italian provenance. This resulted in mean emission rates of 0.56 (GER), 1.33 (ESP) and 1.40 nmol m^−2^ s^−1^ (ITA) after rewetting. *E_S Total_* of the formerly stressed trees was significantly lower in the German than in either the Italian or the Spanish provenance (see Supplementary Table 2a and c available at *Tree Physiology* Online).

In the control groups, *E_S Total_* increased significantly between both sample periods, e.g., for the Italian (increase from 0.60 to 1.16 nmol m^−2^ s^−1^) and the Spanish provenance (increase from 0.78 to 1.68 nmol m^−2^ s^−1^) after the rewetting.

#### Modelling of total isoprenoid emissions

**Figure 3.**
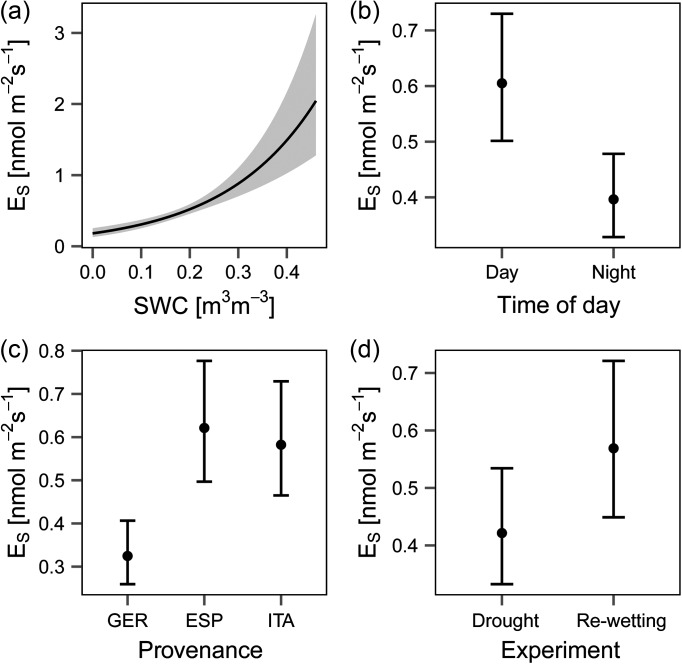
Effect plots of the linear model for total emission rates *E*_*S*_ (normalised to 25 °C by the Guenther 93 algorithm) for (a) SWC, (b) time of day, (c) provenance and (d) sample period. Error bars and ribbons (grey area) represent the standard error and confidence interval, respectively.

A clear relationship between SWC and *E_S Total_* was observed (Figure 3a). Significant effects were found for time of day (Figure 3b), provenance (Figure 3c) and sampling period (Figure 3d) (drought and rewetting.

All provenances showed a significant positive effect of rising SWC levels on *E_S Total_* (Figure 3a). The provenance effect reduced *E_S Total_* by ∼0.29 nmol m^−2^ s^−1^ for the German provenance (Figure 3c) compared with the other provenances. Furthermore, from summer to autumn, *E_S Total_* increased by 0.14 nmol m^−2^ s^−1^ (Figure 3d). Time of day also had an effect on *E_s Total_* with 0.21 nmol m^−2^ s^−1^ higher emission rates during day. The adjusted R² of the final model was 0.50. A simple linear regression approach with *E_s Total_* and SWC is shown in Supplementary Figure S4 available at *Tree Physiology* Online.

### Single compound emission rates

**Figure 4.**
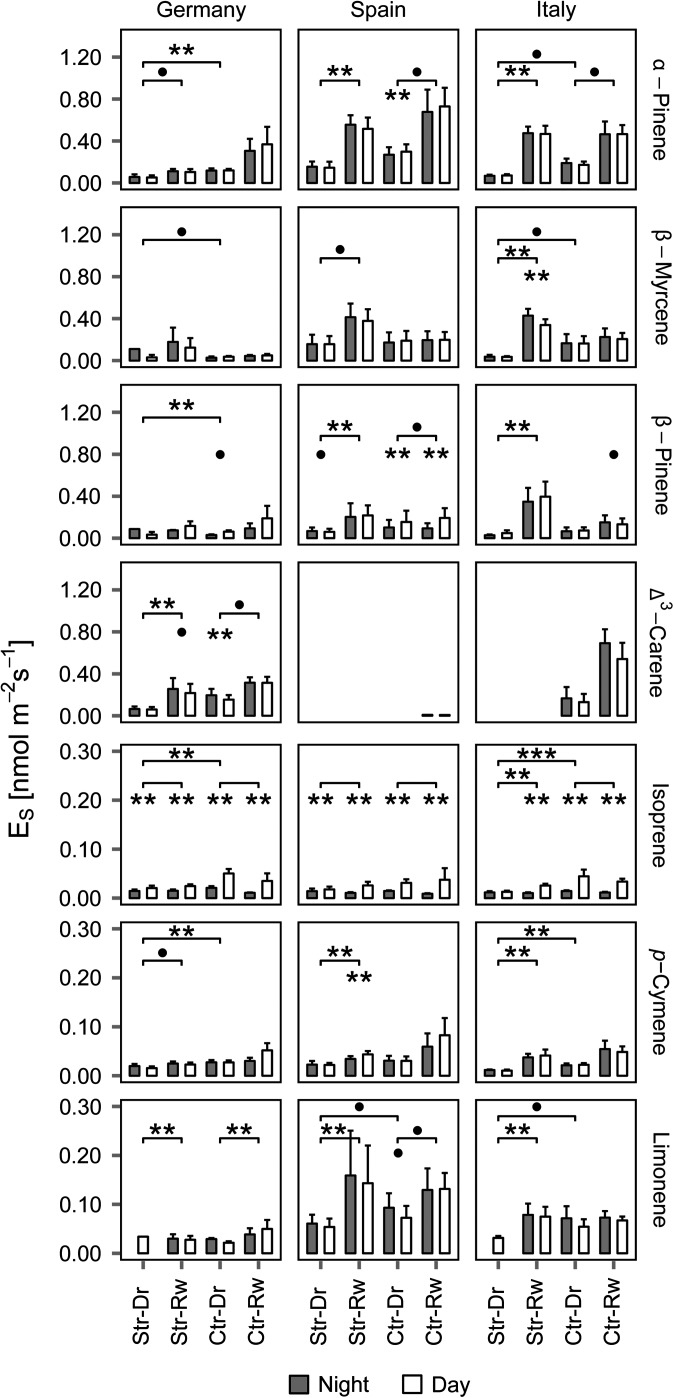
Mean daytime and night-time emission rates (normalised to 25 °C (*E_S_*) by Guenther 93 algorithm) of each quantified compound and provenance separated by treatment and experiment (Str = stressed, Ctr = control, Dr = drought, Rw = rewetting). Bars represent the mean daytime or night-time values of each treatment group and experiment and error bars the standard error. Compounds are ordered in their relative magnitudes (see different scales). Asterisks over bars and brackets represent significant results (***P < 0.01, **P < 0.05, •*P* < 0.1) of the group comparisons in three levels: the lower level shows differences between day and night (Mann–Whitney, paired, above bars), the mid-level between both experiment for control and stressed Mann–Whitney, paired) and top-level differences between stressed and control group during drought sample period (Mann–Whitney, non-paired). *N* = 6 for each group, experiment and time. Further group statistics can be found in Supplementary Tables S3 and S4 available at *Tree Physiology* Online.

The average emission rates (*E_S_*) of the eight quantified compounds largely differed between sample periods, provenances, treatments, and, to a minor degree, between day and night (Figure 4). The trace compound mix 1,8-cineole/*cis*-β-ocimene is emitted by a few trees only and thus is not further discussed here.

**Figure 5.**
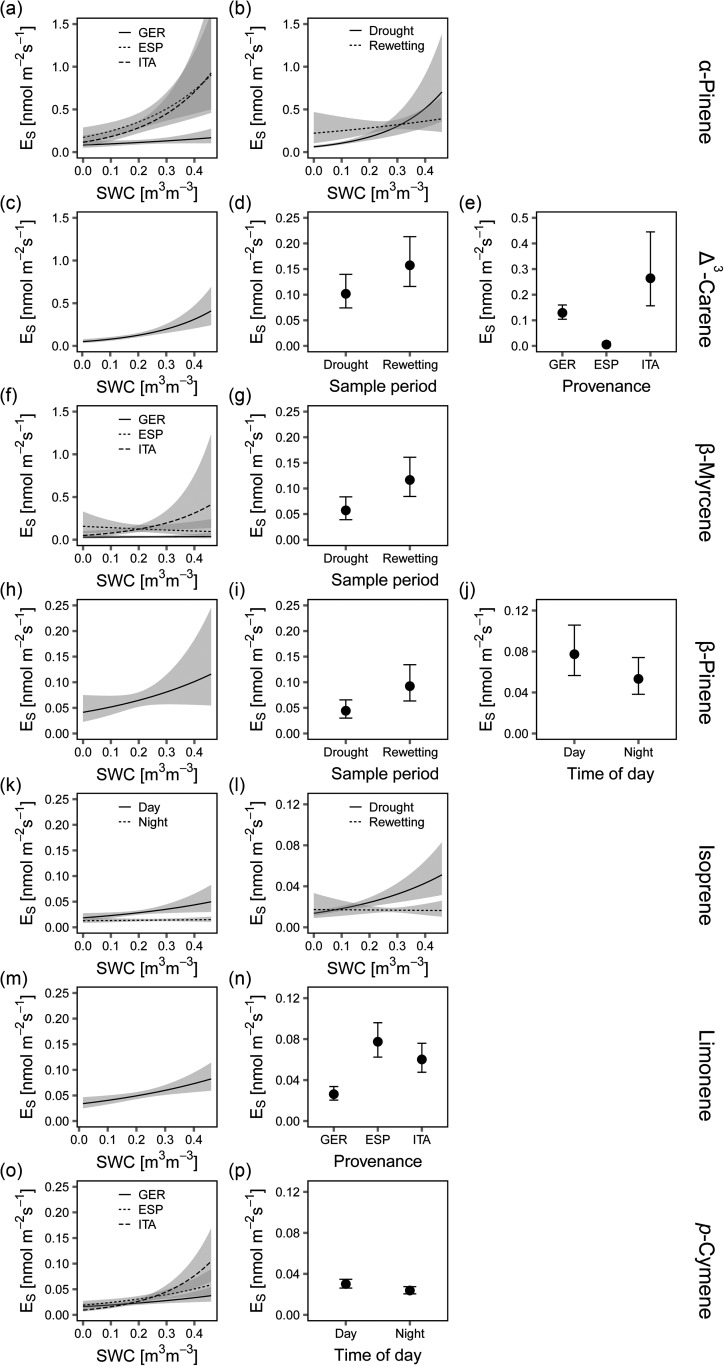
Effect plots for the variables and interactions with volumetric soil water content (SWC) (a, b, f, k, l and o) of the linear models for each single compound emission (variables selected individually for each compound by AIC). Error bars and ribbons represent the standard error and confidence interval, respectively.

The German provenance differed from the other provenances by its throughout abundant high Δ³-carene emissions (see Figure 4). Within the Italian provenance, only two trees were emitting Δ³-carene and none within the Spanish provenance. For the German provenance, no clear treatment effect was observed in summer, however, after rewetting Δ³-carene emissions increased significantly from 0.06 to 0.22 nmol m^−2^ s^−1^ for the stressed and from 0.15 to 0.31 nmol m^−2^ s^−1^ for the control group (see Figure 4). The respective linear model confirmed a positive effect of increasing SWC on Δ³-carene emissions (see Figure 5c). The seasonal effect inherent between the sampling periods was an increase of Δ³-carene emissions by 0.06 nmol m^−2^ s^−1^ after rewetting (see Figure 5d). The significant provenance effect (Figure 5e) confirmed increased Δ³-carene emissions for German and Italian provenances. The adjusted *R*² of the model was 0.60.

Other main emitted compounds were α- and β-pinene as well as β-myrcene (see Figure 4). Significant treatment effects showed lower emissions of these compounds for the German provenance and at a lower significance level also for the Italian, but not for the Spanish provenance. However, comparing drought and rewetting periods, emission rates increased for all groups but with different magnitude and significance for each provenance.

More specifically, α-pinene of the formerly stressed German trees increased only slightly, but significantly, whereas the other two compounds showed no significant changes. Additionally, there was a significant provenance effect: the formerly drought stressed German trees displayed significantly lower α- and β-pinene emissions rates (see Figure 4).

Daytime α- and β-pinene emissions of the stressed Spanish provenance significantly increased after rewetting from 0.14 to 0.52 nmol m^−2^ s^−1^ and from 0.07 to 0.23 nmol m^−2^ s^−1^, respectively. A similar, but less significant increase was measured for the control group. In contrast, daytime β-myrcene emissions significantly increased only for the stressed group (from 0.16 to 0.38 nmol m^−2^ s^−1^) (see Figure 4).

Trees of the stressed Italian provenance significantly increased their α- and β-pinene and β-myrcene emission rates after rewetting from 0.07 to 0.48 nmol m^−2^ s^−1^, from 0.05 to 0.43 nmol m^−2^ s^−1^ and from 0.03 to 0.34 nmol m^−2^ s^−1^, respectively. In the control group, only α-pinene emissions increased significantly from 0.17 to 0.46 nmol m^−2^ s^−1^ after rewetting.

The linear models showed enhanced *E_S_* for α-pinene with increasing SWC. This effect was much stronger for the Spanish and Italian provenances (Figure 5a). The increase of *E_S_* with SWC was stronger after rewetting than during the drought period (Figure 5b). For β-myrcene and β-pinene the SWC influence was much weaker. With season, the emission rates of both compounds increased (Figure 5g and i). Emissions of β-pinene were lower during night (Figure 5j). The adjusted R² of the linear models ranged between 0.55 (α-pinene), 0.32 (β-myrcene) and 0.18 (β-pinene).

Trace compounds in the emission patterns included isoprene, limonene and *p*-cymene. For isoprene, all provenances and treatment groups had a clear night–day pattern with very low emissions during night-time (Figure 4). A significant treatment effect resulted in lower emission rates of the German and Italian provenances at daytime. Only the Italian provenance significantly increased daytime isoprene emission rates after rewetting, from 013 to 0.025 nmol m^−2^ s^−1^. The linear model for isoprene revealed SWC as a significant impact variable, interacting with sample period and day time. Daytime isoprene emission responded to SWC in contrast to night-time emissions (Figure 5k). After rewetting, SWC no longer increased isoprene emission (Figure 5l). The adjusted *R*² was 0.36 (see Supplementary Table S5 available at *Tree Physiology* Online).

The emission rates of limonene strongly differed between the provenances, with the highest rates for the Spanish and the Italian provenances and the lowest for the German one (Figure 4). After rewetting, these significant differences in limonene emission rates were more pronounced. However, rewetting resulted in a strong and (almost throughout) significant increase in limonene emission rates. Daytime limonene emission significantly increased in the stressed groups from 0.06 to 0.14 (ESP) and from 0.03 to 0.08 nmol m^−2^ s^−1^ (ITA). The control groups indicated a few increases after rewetting, significant for the German (0.02 to 0.04 nmol m^−2^ s^−1^) and the Spanish provenances (0.07 to 0.13 nmol m^−2^ s^−1^). The respective linear model equally indicated that limonene emission rates significantly depended on SWC (Figure 5m) as well as on provenance (Figure 5n), with the German provenance emitting significantly least. The adjusted *R*² of the corresponding linear model was 0.30 (see Supplementary Table S5 available at *Tree Physiology* Online).

For *p*-cymene emission, a clear treatment effect was observed for the German and Italian provenances (see Figure 4). Stress trees significantly increased their *p*-cymene emission from 0.02 to 0.04 nmol m^−2^ s^−1^ (ESP) and from 0.01 to 0.04 nmol m^−2^ s^−1^ (ITA) between both sample periods. For *p*-cymene, the corresponding linear model also showed an increase of emission rates with SWC that was different for the provenances (Figure 5o). Additionally, a small, but significant time of day effect was detected with slightly higher emissions during daytime (Figure 5p).

### Relative emitted fraction of isoprenoid compounds and chemo patterns

The mean relative composition of the emitted compounds per tree was grouped into clusters, which were determined for the two sampling periods and day-/night-time. The results are shown in Table 2 (sampling period dry Dr/rewetting Rw and time of day are abbreviated as Dr_Day_, Dr_Night_, Rw_Day_, Rw_Night_).

**Table 2.** Mean relative substance distribution within each cluster.

Cluster type	Lead substance	*N* (Germany)	*N* (Spain)	*N* (Italy)	Period and time of day	α-Pinene	β-Myrcene	β-Pinene	Δ^3^-Carene	Isoprene	1,8-Cineole/*cis-* β-Ocimene	Limonene	*p*-Cymene
1	α : β-Pinene ratio > 4	0	5	4	Dr_Day_	54.3%	16.8%	9.2%	0.0%	7.4%	1.6%	6.4%	4.3%
		0	8	7	Dr_Night_	45.9%	26.4%	12.8%	0.0%	3.5%	0.0%	7.1%	4.4%
		0	5	2	Rw_Day_	59.3%	13.0%	10.1%	0.0%	2.9%	1.9%	9.0%	3.8%
		0	6	2	Rw_Night_	64.2%	12.2%	8.9%	0.1%	1.3%	0.0%	9.5%	3.9%
2	β-Myrcene	0	5	4	Dr_Day_	31.3%	42.8%	6.9%	2.4%	7.7%	0.0%	6.6%	3.6%
		0	2	1	Dr_Night_	7.7%	74.9%	1.2%	8.3%	1.0%	0.0%	5.4%	1.6%
		1	3	4	Rw_Day_	28.1%	46.1%	7.0%	3.6%	2.5%	0.7%	9.7%	2.4%
		1	4	6	Rw_Night_	31.5%	44.1%	9.1%	2.6%	1.0%	0.0%	8.8%	3.0%
3	α : β-Pinene ratio ~ 1	1	2	3	Dr_Day_	32.1%	11.4%	33.8%	3.1%	6.0%	0.0%	8.8%	4.8%
		0	2	3	Dr_Night_	75.3%	4.8%	13.1%	0.0%	4.9%	0.0%	0.0%	4.8%
		1	4	4	Rw_Day_	37.1%	12.9%	35.1%	1.8%	1.8%	0.5%	6.1%	4.6%
		1	2	2	Rw_Night_	39.4%	8.7%	37.1%	3.4%	0.7%	0.2%	7.1%	3.4%
4	Δ3-Carene	11	0	1	Dr_Day_	29.2%	6.9%	7.3%	33.1%	15.6%	0.0%	1.2%	7.1%
		12	0	1	Dr_Night_	32.3%	3.9%	4.6%	45.4%	9.9%	0.0%	0.8%	6.5%
		10	0	2	Rw_Day_	28.6%	8.2%	5.4%	39.4%	6.7%	0.4%	5.1%	6.2%
		10	0	2	Rw_Night_	29.5%	8.8%	4.1%	46.0%	2.8%	0.1%	4.8%	4.9%

Clusters were derived from a PAM cluster analysis based on relative emissions from sample period drought (Dr) and after rewetting (Rw) at both times (Day and Night).

**Figure 6.**
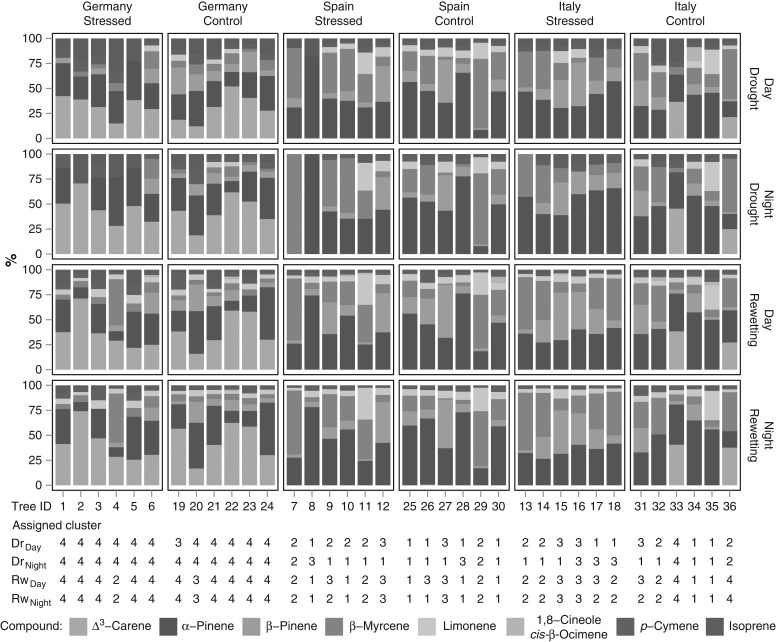
Fractions of quantified compounds per single tree. Compounds under the detection limit were handled as zero values; mean daytime and night-time fractions are shown. Plot splits between each sample period, provenance, treatment and time of day. Tree ID represents each individual measured tree and cluster the determined cluster from Table 2 for each sample period (drought: Dr/rewetting: Rw) and time of day (Day/Night).

In Figure 6 the relative fractions of the compounds emitted by each single tree and the corresponding clusters during night and day are shown.

The four clusters for Dr_Day_, Dr_Night_, Rw_Day_ and Rw_Night_ were matched according to their lead compound: those having a predominant share of Δ^3^-carene (>32%) were summarized in cluster type 4, those predominant in β-myrcene (>42%) in type 2. The remaining ones had predominant shares of α and β-pinene and were summarized in cluster type 1 (ratio α-pinene to β-pinene >4) and in cluster type 3 (ratio α-pinene to β-pinene ∼1). Only the α-pinene to β-pinene ratio emission pattern of Dr_Night_ samples did not fully match these criteria, probably due to extremely low absolute emissions, under detection limit and set to zero, which affected strongly the clustering process. There was a clear effect of provenance on clusters: cluster type 4 mainly consisted of individuals of the German provenance, whereas cluster types 1, 2 and 3 were dominated by Spanish and Italian trees.

In general, the clustering processes for Dr_Day_, Dr_Night_, Rw_Day_ and Rw_Night_ led to different cluster compositions and not all individual trees were assigned to the same cluster type (see Figure 6). However, 52% of the individuals were assigned to the same cluster type in all four cases and 30% of the individuals to the same cluster type in three of four cases. Most of the remaining 18% of individuals were in Dr_Day_, Dr_Night_ or had low SWC contents in the control group.

The share of the different compounds was not constant across clustering for Dr_Day_, Dr_Night_, Rw_Day_ and Rw_Night_. For some compounds, day–night patterns as well as differences between both sample periods were revealed, such as for the isoprene fraction, which was lower during night and after rewetting. The limonene fraction increased from the drought to rewetting in cluster type 1, 2 and 4. After the rewetting, some individuals of the formerly stressed group often emitted additional compounds, such as β-myrcene, β-pinene and limonene, which were below detection limit in summer (see Figure 6).

## Discussion

### Drought stress application

In this experiment potted plants were used instead of containers, because pots allowed an easier handling and randomization of plants within the large-scale experiment. Furthermore, each plant had a clearly defined root space. Related trade-offs, however, were an enhanced soil temperature and, at some point, limitation of plant growth by pot space. For our measurements the mobility aspect and easier handling of the pots was crucial since trees could be moved to the climate chamber with controlled environmental conditions where they were installed into the dynamic-enclosure system for gas exchange studies.

A limitation during the drought experiments was a high variation of SWC in both groups, which had several reasons: first the pre-selected tree seedlings still showed a high variation in size and biomass, mostly due to their provenance-specific adaptation in terms of root and aboveground biomass, needle length as well as shoot/root ratio (see also results of Taeger et al. 2013*a*). Therefore, water uptake by plants may have been uneven within each treatment, which was intensified by different meteorological conditions in the greenhouse of each pre-sampling day. Second, the water supply of each tree differed by a small inherent variation of irrigation by the dripping watering system and the time between the irrigation cycles, e.g. very few control trees of the provenances from Spain and Italy partly experienced some water stress indicated e.g., by SWC values around the wilting point after sampling; however, none of the control trees had SWC values below wilting point before installation. Additionally, during the irrigation cycles of the overlying large-scale experiment, the stressed group had also periodically to be watered in order to reduce the risk of a total plant loss. Finally, SWC measurements by the TDR probe involved uncertainties in terms of integration to a fixed soil volume and averaging across gradients around the dripper. Soil water content could only be measured before and after installation into the plant chambers and therefore pre-installing SWCs were assigned to night-time samples (pre- and post-sampling SWC to both daytime samples). This increased the overall uncertainty of SWC at the point of gas exchange and BVOC measurement.

During the 6 weeks of stress application, SWC of stressed trees was continuously oscillating around the PWP of 0.12 m^3^ m^−3^. The estimation of PWP also included some uncertainties since it was determined in unrooted soil. Roots change pore volume and introduce additional carbon input by dead fine roots, which might alter the soil water regime (Angers and Caron 1998). Therefore, the PWP should rather be seen as a guiding value for the drought application and SWC treatment effects on gas exchange may be less pronounced.

Most importantly, to verify the results of the different group comparisons, potentially restricted by a not throughout perfect SWC separation by the drought treatment, an additional modelling approach based on continuous SWC values was used to cope with this partial stress during summer as well as to identify control variables and to quantify their influence.

#### Net photosynthesis and transpiration

During the long drought stress with reduced SWC, *T*_R_ decreased due to stomatal closure resulting in increasing *T_Leaf_* during daytime. Consequently, *P*_N_ was reduced during stress application. The drought stress was not so severe that it would lead to defoliation due to cavitation or carbon starvation (Salmon et al. 2015), but treated plants showed significantly reduced *P*_N_ and *T*_R_ rates by 60–70% compared with the control group. Although SWC was clearly below the PWP, the ongoing gas exchange implied that plants were still able to extract water from the soil or using water stored in the plant (Zweifel et al. 2007). In addition, the uncertainty regarding regulation and measurement of SWC as well as PWP determination could explain a higher gas exchange than expected. *T_Leaf_* was only moderately enhanced by 2 °C, which corresponded well to leaf-temperature changes reported from other drought experiments such as Ben-Gal et al. (2009) and Scherrer et al. (2011). However, it was obvious that stressed plants reduced their metabolic activity significantly and thus likely had reduced synthesis capacities also for isoprenoids, probably over a longer time scale during the experiment. Among the provenances, the German trees showed higher *P*_N_ and *T*_R_ rates compared with the other two provenances, which related well to a study of Luoma (1997) reporting that trees from their bioclimatic distribution limits show smaller *P*_*N*_ and *T*_R_ due to climatic adaptation under local field conditions. The linear model approach significantly confirmed the strong SWC influence and provenance differences. The interaction of *T*_*Leaf*_ and SWC might be difficult to interpret. However, *T_Leaf_* was clearly related to enhanced photosynthesis activity for SWC above 0.28 m^3^ m^−3^, which is between PWP and field capacity. In contrast, with SWC decreasing below 0.28 m^3^ m^−3^, strongly decreased *T*_R_ and hence *P*_N_ were related to higher *T*_*Leaf*_. The model can, however, only show the behaviour for the measured range, so with a wider range of environmental parameters it is very likely that a saturation point can be modelled.

#### Isoprenoid emissions during drought application

Isoprenoid emissions of Scots pine are generally driven by emission from storage compartments within the plant and to a lesser degree to de novo synthesis of isoprenoids which is temporally linked to photosynthetic activity (faster turn over for isoprene, lagged for MTs) (Steinbrecher et al. 1999, Shao et al. 2001, Ghirardo et al. 2010). Thus, emissions will not fall to zero during longer stress phases or at night. Ghirardo et al. (2010) and Shao et al. (2001) showed with ^13^CO_2_ labelling for Scots pine that some compounds had a higher fraction coming from de novo synthesis instead from storage pools than others, e.g., Δ^3^-carene was emitted less from de novo synthesis compared with α-pinene, β-pinene, camphene, limonene and isoprene. Isoprene is directly coupled to photosynthesis and thus resulted almost completely from de novo synthesis, whereas the other compounds were strongly dependent on the ratio between de novo synthesis and storage pools (Ghirardo et al. 2010). Shao et al. (2001) and Ghirardo et al. (2010) reported that de novo synthesis for MT took several hours and that Scots pine trees were able to synthesize MTs also during night-time from stored carbon, but at a much reduced rate (Shao et al. 2001). In these latter cases, pre-day/-week meteorological and physiological conditions could influence current emission rates since carbon pools used for synthesis are quite variable depending on prior photosynthesis. Furthermore, Ghirardo et al. (2010) observed that a small percentage of de novo synthesized (labelled) monoterpenes were stored in pools.

Based on the above-mentioned findings of the ^13^C labelling studies (Shao et al. 2001, Ghirardo et al. 2010), the influence of drought on the emission rates should differ for single compounds depending on their potential storage capacity and/or de novo synthesis. Compounds with a high de novo ratio should deplete faster due to their smaller pools, whereas compounds emitted predominantly from larger pools could be sustained longer until the storage depletes. This pattern might affect the composition of emission rates of the provenances as indicated by the chemotype clustering. Although drought significantly reduced *E_S Total_*, it did not significantly change the chemotype clustering of the provenances at daytime (see Supplementary Figure S4 available at *Tree Physiology* Online).

The linear models confirmed for almost all compounds that *E_S_* was dependent on SWC, yet to different extents. This suggests that these compounds were partly emitted by de novo synthesis, since SWC reduced the *P*_N_-related synthesis capacity which in turn could lead to reduced *E_S_*. The drought stress also reduced stomatal conductance, yet several studies (e.g. Llusià et al. 1998, Peñuelas and Llusià 1998) indicated that *E*_*s*_ of isoprene and monoterpenes was not affected by stomata closing (Steinbrecher et al. 1997, Niinemets and Reichstein 2003a). Due to the lower stomata conductance the intra leaf gaseous concentration of isoprenoids increases from liquid sources (pool and de novo synthesis) to a new steady state; in this state the higher diffusion gradient increases *E_s_* up to the prior *E_s_* (Steinbrecher et al. 1997, Niinemets and Reichstein 2003*b*).

For another Mediterranean pine (*Pinus halepensis* Mill.) drought-induced decreases in MT emissions were reported by Llusià and Peñuelas (1998) and Blanch et al. (2007).

Day–night differences in monoterpene emission rates were largely not significant. Obviously, isoprene was emitted primarily during daytime which is very well explained by the fact that this compound is not stored and thus is volatized quite fast after synthesis (Ghirardo et al. 2010). The linear models, however, indicated also for β-pinene and *p*-cymene and total emission rate a small time of day effect. For several compounds with a larger contribution by de novo synthesis clear day–night differences would be expected (Steinbrecher et al. 1999). Very few cases of higher or equal night- versus daytime emissions might have been induced by mechanical stress or damage of needles, despite careful tree handling. Since the whole upper part of the trees was placed in the enclosure, emissions exclusively from pools in the bark and stem might have reduced differences between day and night. Since emission rates were normalized to leaf area and 25 °C, these non-leaf compartments and thermal correction could have introduced some uncertainties.

### Recovery phase/rewetting

#### Net photosynthesis and transpiration

After the 6-week recovery phase under normal water regime, a significantly higher SWC was observed and considered as well-watered. Control SWC was higher than in summer, since temperature und radiation in the greenhouse were lower. However, SWC of the stressed groups was lower than the respective controls because the formerly dried-out substrate tended to be hydrophobic; nevertheless, field capacity was reached. Therefore, *T*_R_ and *P*_N_ of the stressed groups increased with a concomitant decrease of *T_Leaf_* to similar levels compared with their controls, indicating a recovery from drought. Compared with the drought sample period, the control groups of the German and Spanish provenances had a reduced *P*_N_, whereas the *P*_N_ of the Italian provenance stayed at the same level. This was additionally confirmed by the linear models showing on average lower *P*_N_ and *T*_R_ for the rewetting period. It is likely that this seasonal variation of *P*_N_ is an adaptation to local climates of each provenance (Luoma 1997) and/or seasonal development (Vogg et al. 1998) leading to lower gas exchange rates in autumn due to leaf aging (Freeland 1952).

#### Isoprenoid emissions after rewetting

Impacts of drought stress on isoprenoid emission were not only revealed by treatment effects during the drought, but also by differences between both sampling periods, when the emission rates of the same individuals were re-measured after rewetting for 6 weeks. Here, an increase in emissions of all compounds could be observed for stressed and control trees; however, for the formerly stressed trees, this increase was more pronounced, pointing to a clear recovery from drought stress.

The higher emission rates of the stressed trees after 6 weeks rewetting corresponded well to the increased net photosynthesis, which indicated an increased carbon allocation. This additional carbon was then also invested for isoprenoid synthesis, which could be used in refilling the storage pools (e.g., during the recovery time) and also supported de novo emissions. Considering that the control trees emitted less isoprenoids during the drought despite a higher *P*_N_ than after recovery, it may be assumed that further ongoing MT synthesis filled up pools. A possible reason for the increase of emission rates could be due to the greenhouse conditions, e.g., through higher ambient temperature and lower diurnal temperature differences. These optimized growing conditions might have further enhanced the synthesis capability for isoprenoid production and storage increase also towards autumn. Linear models clearly confirmed the strong dependency of emission rates on SWC linked to seasonal effects for many compounds. Turtola et al. (2003) showed that drought increased resin content in woody parts for drought stressed Scots pine seedlings; this can probably cause more emissions. A general seasonal influence has been observed in other studies, e.g., increased monoterpene content in needles in autumn for *P. halepensis* (Llusià and Peñuelas 2000) or for *Pinus banksiana* Lamb. (Lerdau et al. 1997). Under natural conditions in Finland Räisänen et al. (2009) reported a slightly decreasing MT content for current *Pinus sylvestris* L. needles towards the end of the growing season. Emission rates of Scots pine under field conditions decreased till the end of the growing season, mostly related to lower radiation and temperature (Komenda 2002, Holzke et al. 2006*a*). Therefore, location and environmental condition as well as provenance might have a strong effect on the base emission capability of isoprenoids from Scots pine.

Drought impacts and seasonal development were also apparent in the relative isoprenoid compound fractions where stressed and control trees both shifted their emission patterns. This was indicated by the compound distribution within each cluster type. However, a clear separation of recovery effects from drought stress and seasonal change effects was difficult, since pre-drought measurements were not possible due to system constraints. Yet, seasonal shifts in Scots pine isoprenoid patterns were observed by several studies (e.g., Komenda 2002, Tarvainen et al. 2005, Räisänen et al. 2009). Komenda (2002) showed for example for β-myrcene, *p*-cymene or limonene ratios a decrease over summer and a recovery in autumn corresponding to our seasonal pattern in the control groups.

Each provenance displayed enhanced emission rates after rewetting, but diverging in strength and at total and single compound level. The two southern provenances invested more in additional isoprenoids during autumn, whereas for the German provenance these changes were only moderate. Possible reasons for this provenance-specific seasonal adaptation of the isoprenoid amounts could be related to their defence function against insect feeding. Ruby and Wright (1976) revealed that impacts of herbivore species attacks differed between provenances; in particular, northern provenances were more endangered than Mediterranean ones. It is well known that increasing terpene concentrations within needles reduces herbivore feeding (Manninen et al. 2002, O'Reilly-Wapstra et al. 2007, Iason et al. 2011). Thus, their findings are in line with our study reporting a smaller autumnal increase for the northern-most German than for the Spanish and Italian provenances. We hypothesize that Mediterranean provenances have to be adapted to possible insect attacks during their (relatively warm) winter, thus building up a high capacity for MT emissions.

### Chemotypes and emission patterns

Tobolski and Hanover (1971) identified distinct chemotypes in Scots pine from Europe by analysing needle resin. Our study is, to our knowledge, the first one supporting this classification by directly measuring emission patterns of Scots pine provenances from Spain, Italy and Germany.

The main emitted compounds α-pinene, β-pinene, β-myrcene and Δ³-carene had the highest discriminative power in the cluster analysis. Trees from Spain showed no Δ³-carene emissions and only 2 out of 12 trees of the Italian provenance emitted Δ³-carene. These non Δ³-carene emitters can be further split into three groups depending on their α- to β-pinene ratio and β-myrcene fraction. In contrast, the German provenance was a Δ³-carene-dominated type. The observed isoprenoid emission fractions in autumn corresponded well to the resin analysis conducted by Tobolski and Hanover (1971) showing a typical Δ³-carene and non-Δ³-carene separation between the provenances. A pronounced different day to night emission pattern was only visible for isoprene, which was more coupled to photosynthesis (Ghirardo et al. 2010). During the drought application some compounds were absent or reduced, e.g. limonene or β-myrcene/-pinene. During Dr_Night_ several individuals emitted compounds below their detection limit, which were further handled as zero values, resulting in different cluster compositions.

Compound fractions varied considerably among single trees of a provenance, which might be related to genetic variation shown for example within stands (Bäck et al. 2012, Kannaste et al. 2013) and between different trees (e.g., Komenda 2002, Komenda et al. 2003, Holzke et al. 2006*b*, Yassaa et al. 2012).

## Conclusions

In relation to the whole-plant carbon uptake and usage, the loss due to VOC emission is normally a few percent (e.g., Kesselmeier and Staudt 1999). This carbon is invested for synthesizing ecological relevant compounds used in plant–plant as well as in plant–insect interactions. Furthermore, they are important players in atmospheric chemistry. Thus, even changes in low-trace compounds might be important independent of their physiological and/or ecological purpose.

This study showed that Scots pine provenances consisted of different chemotypes, classified in four groups at the end of the growing season as Δ³-carene emitters (GER: 10 out of 12 trees; ITA: 2 out of 12 trees) and non-Δ³-carene emitters which are either β-myrcene dominated (GER: 1; ESP: 4; ITA: 6) or are separated by their α-/β-pinene emission ratios (GER: 1; ESP: 8; ITA: 4). Their isoprenoid ‘bouquet’ changed little from summer to autumn. Thus, the first hypothesis that chemotype patterns of Scots pine seedlings differ among and within provenances, but individual fingerprints remain also under different treatments, has to be accepted. However, drought stress impacted the chemotype composition in such a manner that a few compounds were below the detection limit. It is idle to argue whether the drought stress applied was severe or medium level, nevertheless, it led to a reduced gas exchange and enhanced leaf temperatures as well as reducing emission rates. The stressed trees were able to recover from drought and reached gas exchange levels of the respective control groups after rewetting. Thus, also the second hypothesis that Scots pine isoprenoid emissions change during and after long-term drought stress, but can recover to non-stressed (normal) levels, can be accepted. Finally, isoprenoid emission rates differed between provenances in strength as well as in plasticity to recover from longer stress application (third hypothesis), yet, it is difficult to determine how this is linked to apparent morphological differences. The strong SWC dependency in the respective linear models also supports this last hypothesis.

Chemotypes and isoprenoid emission rates differed between provenances and treatments, which will affect their temporal and spatial abundance. This is important for atmospheric chemical processes, since reactivity and lifetime of isoprenoid compounds vary depending on potential reaction partners (e.g., O_3,_ OH or NO_3_ radical) (Atkinson and Arey 2003). Additionally, processes in atmospheric chemistry strongly depend on solar radiation and air temperature, which are usually higher during drought periods. However, under these conditions Scots pine isoprenoid emission, thus biogenic precursor compounds for air pollutants, are not necessarily increased. Therefore, it is important to consider this fact in BVOC emission and atmospheric chemistry models on different temporal and spatial scales. For local stands, an impact on atmospheric chemistry processes by different emitting trees has already been shown (Smolander et al. 2014).

We are aware that our database on isoprenoid measurements from different provenances is relatively small. A much larger statistical sample size would be necessary to provide complete provenance-specific and/or chemotype-corrected isoprenoid emission rates needed for the different applications. Further experiments on Scots pine should include a higher temporal resolution of isoprenoid sampling, especially before and during drought application, and comprise other provenances. A clear separation between wood and leaf emission could help for better interpretation of drought stress effects.

## Supplementary data

Supplementary data for this article are available at *Tree Physiology* Online.

## Funding

The research leading to these results has received funding from the European Research Council under the European Union's Seventh Framework Programme (FP7/2007-2013)/ERC grant agreement No. (282250). Funding for travel und conference costs by MICMoR (Mechanisms and Interactions of Climate Change in Mountain Regions Helmholtz Research School) is appreciated. The study was performed with the support of the Technische Universität München – Institute for Advanced Study, funded by the German Excellence Initiative. Special thanks to Barbara Weber, who supported this study with her Bachelor Thesis.

## References

[tpw066C1] Achotegui-Castells A, Llusià J, Hódar JA, Peñuelas J (2013) Needle terpene concentrations and emissions of two coexisting subspecies of Scots pine attacked by the pine processionary moth (*Thaumetopoea pityocampa*). Acta Physiol Plant 35:3047–3058.

[tpw066C2] Angers DA, Caron J (1998) Plant-induced changes in soil structure: processes and feedbacks. Biogeochemistry 42:55–72.

[tpw066C3] Arneth A, Monson RK, Schurgers G, Niinemets Ü, Palmer PI (2008) Why are estimates of global terrestrial isoprene emissions so similar (and why is this not so for monoterpenes)?. Atmos Chem Phys 8:4605–4620.

[tpw066C4] Atkinson R, Arey J (2003) Gas-phase tropospheric chemistry of biogenic volatile organic compounds: a review. Atmos Environ 37:197–219.

[tpw066C6] Bäck J, Hari P, Hakola H, Juurola E, Kulmala M (2005) Dynamics of monoterpene emissions in *Pinus sylvestris* during early spring. Boreal Env Res 10:409–424.

[tpw066C5] Bäck J, Aalto J, Henriksson M, Hakola H, He Q, Boy M (2012) Chemodiversity of a Scots pine stand and implications for terpene air concentrations. Biogeosciences 9:689–702.

[tpw066C7] Ben-Gal A, Agam N, Alchanatis V, Cohen Y, Yermiyahu U, Zipori I, Presnov E, Sprintsin M, Dag A (2009) Evaluating water stress in irrigated olives: correlation of soil water status, tree water status, and thermal imagery. Irrig Sci 27:367–376.

[tpw066C8] Blanch J, Peñuelas J, Llusià J (2007) Sensitivity of terpene emissions to drought and fertilization in terpene-storing *Pinus halepensis* and non-storing *Quercus ilex*. Physiol Plant 131:211–225. 18251893 10.1111/j.1399-3054.2007.00944.x

[tpw066C9] Blanch J, Peñuelas J, Sardans J, Llusià J (2009) Drought, warming and soil fertilization effects on leaf volatile terpene concentrations in *Pinus halepensis* and *Quercus ilex*. Acta Physiol Plant 31:207–218.

[tpw066C10] von Caemmerer S, Farquhar GD (1981) Some relationships between the biochemistry of photosynthesis and the gas exchange of leaves. Planta 153:376–387. 24276943 10.1007/BF00384257

[tpw066C11] Dindorf T, Kuhn U, Ganzeveld L, Schebeske G, Ciccioli P, Holzke C, Köble R, Seufert G, Kesselmeier J (2006) Significant light and temperature dependent monoterpene emissions from European beech (*Fagus sylvatica L.*) and their potential impact on the European volatile organic compound budget. J Geophys Res 111:137–182.

[tpw066C12] Felzer BS, Cronin T, Reilly JM, Melillo JM, Wang X (2007) Impacts of ozone on trees and crops. C R Geosci 339:784–798.

[tpw066C13] Freeland RO (1952) Effect of age of leaves upon the rate of photosynthesis in some conifers. Plant Physiol 27:685–690. 16654494 10.1104/pp.27.4.685 PMC547980

[tpw066C14] Galiano L, Martínez-Vilalta J, Lloret F (2010) Drought-induced multifactor decline of scots pine in the pyrenees and potential vegetation change by the expansion of co-occurring oak species. Ecosystems 13:978–991.

[tpw066C15] Ghirardo A, Koch K, Taipale R, Zimmer IN, Schnitzler J, Rinne J (2010) Determination of de novo and pool emissions of terpenes from four common boreal/alpine trees by ^13^CO_2_ labelling and PTR-MS analysis. Plant Cell Environ 33:781–792. 20040067 10.1111/j.1365-3040.2009.02104.x

[tpw066C16] Grote R, Keenan T, Lavoir A, Staudt M (2010) Process-based simulation of seasonality and drought stress in monoterpene emission models. Biogeosciences 7:257–274.

[tpw066C17] Guenther A, Zimmerman PR, Harley PC, Monson RK, Fall R (1993) Isoprene and monoterpene emission rate variability: Model evaluations and sensitivity analyses. J Geophys Res 98:12609.

[tpw066C18] Guenther A, Hewitt CN, Erickson D et al (1995) A global model of natural volatile organic compound emissions. J Geophys Res 100:8873.

[tpw066C19] Guenther AB, Jiang X, Heald CL, Sakulyanontvittaya T, Duhl T, Emmons LK, Wang X (2012) The model of emissions of gases and aerosols from nature version 2.1 (MEGAN2.1): an extended and updated framework for modeling biogenic emissions. Geosci Model Dev 5:1471–1492.

[tpw066C20] Haase KB, Jordan C, Mentis E, Cottrell L, Mayne HR, Talbot R, Sive BC (2011) Changes in monoterpene mixing ratios during summer storms in rural New Hampshire (USA). Atmos Chem Phys 11:11465–11476.

[tpw066C21] Hakola H, Tarvainen V, Back J, Ranta H, Bonn B, Rinne J, Kulmala M (2006) Seasonal variation of mono- and sesquiterpene emission rates of Scots pine. Biogeosciences 3:93–101.

[tpw066C22] Holzke C, Dindorf T, Kesselmeier J, Kuhn U, Koppmann R (2006 *a*) Terpene emissions from European beech (*Fagus sylvatica L.*): pattern and emission behaviour over two vegetation periods. J Atmos Chem 55:81–102.

[tpw066C23] Holzke C, Hoffmann T, Jaeger L, Koppmann R, Zimmer W (2006 *b*) Diurnal and seasonal variation of monoterpene and sesquiterpene emissions from Scots pine (*Pinus sylvestris L*.). Atmos Environ 40:3174–3185.

[tpw066C24] Iason GR, O'Reilly-Wapstra JM, Brewer MJ, Summers RW, Moore BD (2011) Do multiple herbivores maintain chemical diversity of Scots pine monoterpenes?. Philos T R Soc B 366:1337–1345. 10.1098/rstb.2010.0236 PMC3081569 21444308

[tpw066C25] Kannaste A, Copolovici L, Pazouki L, Suhhorutsenko M, Niinemets U (2013) Highly variable chemical signatures over short spatial distances among Scots pine (*Pinus sylvestris*) populations. Tree Physiol 33:374–387. 23513034 10.1093/treephys/tpt013

[tpw066C26] Kesselmeier J, Staudt M (1999) Biogenic volatile organic compounds (VOC): an overview on emission, physiology and ecology. J Atmos Chem 33:23–88.

[tpw066C27] Klepzig KD, Kruger EL, Smalley EB, Raffa KF (1995) Effects of biotic and abiotic stress on induced accumulation of terpenes and phenolics in red pines inoculated with bark beetle-vectored fungus. J Chem Ecol 21:601–626. 24234253 10.1007/BF02033704

[tpw066C28] Komenda M (2002) Monoterpene emissions from Scots pine (*Pinus sylvestris*): field studies of emission rate variabilities. J Geophys Res 107:4161.

[tpw066C29] Komenda M, Kobel K, Koppmann R, Wildt J (2003) Comparability of biogenic VOC emission rate measurements under laboratory and ambient conditions at the example of monoterpene emissions from Scots pine (*Pinus sylvestris*). J Atmos Chem 45:1–23.

[tpw066C30] Kulmala M, Suni T, Lehtinen KEJ et al (2004) A new feedback mechanism linking forests, aerosols, and climate. Atmos Chem Phys 4:557–562.

[tpw066C31] Langenheim JH (1994) Higher plant terpenoids: a phytocentric overview of their ecological roles. J Chem Ecol 20:1223–1280. 24242340 10.1007/BF02059809

[tpw066C32] Lerdau M, Matson P, Fall R, Monson R (1995) Ecological controls over monoterpene emissions from Douglas-Fir (*Pseudotsuga Menziesii*). Ecology 76:2640–2647.

[tpw066C33] Lerdau M, Litvak M, Palmer P, Monson R (1997) Controls over monoterpene emissions from boreal forest conifers. Tree Physiol 17:563–569. 14759829 10.1093/treephys/17.8-9.563

[tpw066C34] Llusià J, Peñuelas J (1998) Changes in terpene content and emission in potted Mediterranean woody plants under severe drought. Can J Bot 76:1366–1373.

[tpw066C35] Llusià J, Peñuelas J (2000) Seasonal patterns of terpene content and emission from seven Mediterranean woody species in field conditions. Am J Bot 87:133–140. 10636836

[tpw066C36] Loreto F, Schnitzler J (2010) Abiotic stresses and induced BVOCs. Trends Plant Sci 15:154–166. 20133178 10.1016/j.tplants.2009.12.006

[tpw066C37] Luoma S (1997) Geographical pattern in photosynthetic light response of *Pinus sylvestris* in Europe. Funct Ecol 11:273–281.

[tpw066C38] Manninen A, Tarhanen S, Vuorinen M, Kainulainen P (2002) Comparing the variation of needle and wood terpenoids in Scots pine provenances. J Chem Ecol 28:211–228. 11868675 10.1023/a:1013579222600

[tpw066C39] Niinemets Ü (2010) Mild versus severe stress and BVOCs: thresholds, priming and consequences. Trends Plant Sci 15:145–153. 20006534 10.1016/j.tplants.2009.11.008

[tpw066C40] Niinemets Ü, Reichstein M (2003 *a*) Controls on the emission of plant volatiles through stomata: a sensitivity analysis. J Geophys Res 108(D7):4211, doi:10.1029/2002JD002626.

[tpw066C41] Niinemets Ü, Reichstein M (2003 *b*) Controls on the emission of plant volatiles through stomata: differential sensitivity of emission rates to stomatal closure explained. J Geophys Res 108(D7):4208, doi:10.1029/2002JD002620.

[tpw066C42] Niinemets Ü, Loreto F, Reichstein M (2004) Physiological and physicochemical controls on foliar volatile organic compound emissions. Trends Plant Sci 9:180–186. 15063868 10.1016/j.tplants.2004.02.006

[tpw066C43] Niinemets Ü, Kuhn U, Harley PC et al (2011) Estimations of isoprenoid emission capacity from enclosure studies: measurements, data processing, quality and standardized measurement protocols. Biogeosciences 8:2209–2246.

[tpw066C44] Niinemets Ü, Fares S, Harley P, Jardine KJ (2014) Bidirectional exchange of biogenic volatiles with vegetation: emission sources, reactions, breakdown and deposition. Plant Cell Environ 37:1790–1809. 24635661 10.1111/pce.12322 PMC4289707

[tpw066C45] Oleksyn J, Tjoelker M, Reich P (1998) Adaptation to changing environment in Scots pine populations across a latitudinal gradient. Silva Fenn 32:129–140.

[tpw066C46] Ortega J, Helmig D (2008) Approaches for quantifying reactive and low-volatility biogenic organic compound emissions by vegetation enclosure techniques – Part A. Chemosphere 72:343–364. 18279913 10.1016/j.chemosphere.2007.11.020

[tpw066C47] O'Reilly-Wapstra JM, Iason GR, Thoss V (2007) The role of genetic and chemical variation of *Pinus sylvestris* seedlings in influencing slug herbivory. Oecologia 152:82–91. 17180371 10.1007/s00442-006-0628-4

[tpw066C48] Pearson M, Saarinen M, Nummelin L et al (2013) Tolerance of peat-grown Scots pine seedlings to waterlogging and drought: Morphological, physiological, and metabolic responses to stress. Forest Ecol Manag 307:43–53.

[tpw066C49] Peñuelas J, Llusià J (1999) Seasonal emission of monoterpenes by the Mediterranean tree *Quercus ilex* in field conditions: Relations with photosynthetic rates, temperature and volatility. Physiol Plant 105:641–647.

[tpw066C50] Peñuelas J, Staudt M (2010) BVOCs and global change. Trends Plant Sci 15:133–144. 20097116 10.1016/j.tplants.2009.12.005

[tpw066C51] Perakyla O, Vogt M, Tikkanen O et al (2014) Monoterpenes’ oxidation capacity and rate over a boreal forest: temporal variation and connection to growth of newly formed particles. Boreal Env Res 19:293–310.

[tpw066C52] Pierce JR, Leaitch WR, Liggio J et al (2012) Nucleation and condensational growth to CCN sizes during a sustained pristine biogenic SOA event in a forested mountain valley. Atmos Chem Phys 12:3147–3163.

[tpw066C53] Plewka A, Gnauk T, Brüggemann E, Herrmann H (2006) Biogenic contributions to the chemical composition of airborne particles in a coniferous forest in Germany. Atmos Environ 40:103–115.

[tpw066C55] Räisänen T, Ryyppö A, Kellomäki S (2009) Monoterpene emission of a boreal Scots pine (*Pinus sylvestris L.*) forest. Agr Forest Meteorol 149:808–819.

[tpw066C54] R Development Core Team (2014) R: a language and environment for statistical computing. R Foundation for Statistical Computing, Vienna, Austria. http://www.R-project.org.

[tpw066C56] Rebetez M, Dobbertin M (2004) Climate change may already threaten Scots pine stands in the Swiss Alps. Theor Appl Climatol 79:1–9.

[tpw066C57] Reich PB, Oleksyn J (2008) Climate warming will reduce growth and survival of Scots pine except in the far north. Ecol Lett 11:588–597. 18363717 10.1111/j.1461-0248.2008.01172.x

[tpw066C58] Ruby JL, Wright JW (1976) A revised classification of geographic varieties in Scots pine. Silvae Genet 25(5–6):169–175.

[tpw066C59] Sallas L, Luomala EM, Utriainen J, Kainulainen P, Holopainen JK (2003) Contrasting effects of elevated carbon dioxide concentration and temperature on Rubisco activity, chlorophyll fluorescence, needle ultrastructure and secondary metabolites in conifer seedlings. Tree Physiol 23:97–108. 12533304 10.1093/treephys/23.2.97

[tpw066C60] Salmon Y, Torres-Ruiz JM, Poyatos R, Martinez-Vilalta J, Meir P, Cohard H, Mencuccini M, Cochard H (2015) Balancing the risks of hydraulic failure and carbon starvation: a twig scale analysis in declining Scots pine. Plant Cell Environ 38:2575–2588. 25997464 10.1111/pce.12572 PMC4989476

[tpw066C61] Schade GW, Solomon SJ, Dellwik E, Pilegaard K, Ladstätter-Weissenmayer A (2011) Methanol and other VOC fluxes from a Danish beech forest during late springtime. Biogeochemistry 106:337–355.

[tpw066C62] Scherrer D, Bader MK, Körner C (2011) Drought-sensitivity ranking of deciduous tree species based on thermal imaging of forest canopies. Agr Forest Meteorol 151:1632–1640.

[tpw066C63] Schneider CA, Rasband WS, Eliceiri KW (2012) NIH Image to ImageJ: 25 years of image analysis. Nat Meth 9:671–675. 10.1038/nmeth.2089 PMC5554542 22930834

[tpw066C64] Scott CE, Rap A, Spracklen DV et al (2014) The direct and indirect radiative effects of biogenic secondary organic aerosol. Atmos Chem Phys 14:447–470.

[tpw066C65] Shao M, Czapiewski KV, Heiden AC, Kobel K, Komenda M, Koppmann R, Wildt J (2001) Volatile organic compound emissions from Scots pine: Mechanisms and description by algorithms. J Geophys Res 106:20483–20491.

[tpw066C66] Šimpraga M, Verbeeck H, Demarcke M et al (2011) Clear link between drought stress, photosynthesis and biogenic volatile organic compounds in *Fagus sylvatica L*. Atmos Environ 45:5254–5259.

[tpw066C67] Smolander S, He Q, Mogensen D et al (2014) Comparing three vegetation monoterpene emission models to measured gas concentrations with a model of meteorology, air chemistry and chemical transport. Biogeosciences 11:5425–5443.

[tpw066C68] Spinelli F, Cellini A, Marchetti L, Mudigere K, Piovene C (2011) Emission and function of volatile organic compounds in response to abiotic stress In: Shanker A (ed.) Abiotic stress in plants - mechanisms and adaptations. Rijeka, Croatia, pp 367–394.

[tpw066C69] Staudt M, Joffre R, Rambal S (2003) How growth conditions affect the capacity of *Quercus ilex* leaves to emit monoterpenes. New Phytol 158:61–73.

[tpw066C70] Stein S (2008) NIST standard reference database 1A. National Institute of Standards and Technology, Gaithersburg.

[tpw066C71] Steinbrecher R, Hauff K, Rabong R, Steinbrecher J (1997) Isoprenoid emission of oak species typical for the Mediterranean area: source strenght and controlling variables. Atmos Environ 31:79–88.

[tpw066C72] Steinbrecher R, Hauff K, Hakola H, Rössler J (1999) A revised parameterisation for emission modelling of isoprenoids for boreal plants In: Laurila T, Lindfors V (eds) BiogenicVOC emissions and photochemistry in the boreal regions of Europe. Commission of European Communities, Luxembourg, pp 29–43.

[tpw066C73] Stevenson DS, Young PJ, Naik V et al (2013) Tropospheric ozone changes, radiative forcing and attribution to emissions in the Atmospheric Chemistry and Climate Model Intercomparison Project (ACCMIP). Atmos Chem Phys 13:3063–3085.

[tpw066C74] Taeger S, Fussi B, Konnert M, Menzel A (2013 *a*) Large-scale genetic structure and drought-induced effects on European Scots pine (*Pinus sylvestris L.*) seedlings. Eur J Forest Res 132:481–496.

[tpw066C75] Taeger S, Zang C, Liesebach M, Schneck V, Menzel A (2013 *b*) Impact of climate and drought events on the growth of Scots pine (*Pinus sylvestris L.*) provenances. For Ecol Manag 307:30–42.

[tpw066C76] Tarvainen V, Hakola H, Hellen H, Back J, Hari P, Kulmala M (2005) Temperature and light dependence of the VOC emissions of Scots pine. Atmos Chem Phys 5:989–998.

[tpw066C77] Thoss V, O'Reilly-Wapstra J, Iason GR (2007) Assessment and Implications of intraspecific and phenological variability in monoterpenes of Scots Pine (*Pinus sylvestris*) Foliage. J Chem Ecol 33:477–491. 10.1007/s10886-006-9244-3 17268824

[tpw066C78] Tobolski JJ, Hanover JW (1971) Genetic variation in monoterpenes of Scotch pine. Forest Sci 17:293–299.

[tpw066C79] Turtola S, Manninen A, Rikala R, Kainulainen P (2003) Drought stress alters the concentration of wood terpenoids in Scots Pine and Norway Spruce seedlings. J Chem Ecol 29:1981–1995. 14584671 10.1023/a:1025674116183

[tpw066C80] Vogg G, Heim R, Hansen J, Schäfer C, Beck E (1998) Frost hardening and photosynthetic performance of Scots pine (*Pinus sylvestris L*.) needles. I. Seasonal changes in the photosynthetic apparatus and its function. Planta 204:193–200.

[tpw066C81] Wickham H (2009) ggplot2: elegant graphics for data analysis. Springer, New York, NY.

[tpw066C82] Yassaa N, Song W, Lelieveld J, Vanhatalo A, Bäck J, Williams J (2012) Diel cycles of isoprenoids in the emissions of Norway spruce, four Scots pine chemotypes, and in Boreal forest ambient air during HUMPPA-COPEC-2010. Atmos Chem Phys Discuss 12:10425–10460.

[tpw066C83] Zweifel R, Steppe K, Sterck FJ (2007) Stomatal regulation by microclimate and tree water relations: interpreting ecophysiological field data with a hydraulic plant model. J Exp Bot 58:2113–2131. 17490998 10.1093/jxb/erm050

